# Biosynthesis of Palladium Nanoparticles by Using Aqueous Bark Extract of *Quercus dalechampii*, *Q. frainetto,* and *Q. petraea* for Potential Antioxidant and Antimicrobial Applications

**DOI:** 10.3390/plants13233390

**Published:** 2024-12-03

**Authors:** Nastaca-Alina Coman, Mihai Babotă, Alexandru Nicolescu, Alexandra Nicolae-Maranciuc, Lavinia Berta, Adrian Man, Dan Chicea, Lenard Farczadi, Corneliu Tanase

**Affiliations:** 1 Doctoral School of Medicine and Pharmacy, “George Emil Palade“ University of Medicine, Pharmacy, Science, and Technology of Targu Mures, 540142 Targu Mures, Romania; nastaca-alina.coman@umfst.ro; 2 Research Center of Medicinal and Aromatic Plants, “George Emil Palade” University of Medicine, Pharmacy, Sciences and Technology of Targu Mures, 540139 Targu Mures, Romania; corneliu.tanase@umfst.ro; 3 Department of Pharmaceutical Botany, Faculty of Pharmacy, “George Emil Palade” University of Medicine, Pharmacy, Sciences and Technology of Targu Mures, 540139 Targu Mures, Romania; 4 Laboratory of Chromatography, Institute of Advanced Horticulture Research of Transylvania, Faculty of Horticulture and Business in Rural Development, University of Agricultural Sciences and Veterinary Medicine, 400372 Cluj-Napoca, Romania; alexandru.nicolescu@usamvcluj.ro; 5 Research Center for Complex Physical Systems, Faculty of Sciences, Lucian Blaga University of Sibiu, 550012 Sibiu, Romania; alexandra.nicolae@ulbsibiu.ro (A.N.-M.); dan.chicea@ulbsibiu.ro (D.C.); 6 Institute for Interdisciplinary Studies and Research (ISCI), Lucian Blaga University of Sibiu, 550024 Sibiu, Romania; 7 Department of General and Inorganic Chemistry, “George Emil Palade” University of Medicine, Pharmacy, Sciences and Technology of Târgu Mures, 540139 Targu Mures, Romania; lavinia.berta@umfst.ro; 8 Department of Microbiology, Faculty of Medicine, “George Emil Palade” University of Medicine, Pharmacy, Sciences and Technology of Targu Mures, 540142 Targu Mures, Romania; adrian.man@umfst.ro; 9 Chromatography and Mass Spectrometry Laboratory, Center for Advanced Medical and Pharmaceutical Research, “George Emil Palade” University of Medicine, Pharmacy, Sciences and Technology of Targu Mures, 540139 Targu Mures, Romania; lenard.farczadi@umfst.ro

**Keywords:** biosynthesis, palladium nanoparticles, *Quercus*, rhytidome, phenolic compounds, antioxidant, antimicrobial

## Abstract

This study aimed to synthesize palladium nanoparticles (PdNPs) using bioactive compounds from aqueous extracts of *Quercus* species (*Quercus dalechampii*, *Quercus frainetto*, and *Quercus petraea*) with potential biomedical applications. To optimize PdNPs biosynthesis, various parameters were explored, including the concentration of PdCl_2_, the extract-to-PdCl_2_ ratio, and the pH of the solution. The nanoparticles were characterized using ultraviolet/visible spectroscopy (UV/Vis), Fourier-transform infrared spectroscopy (FTIR), and dynamic light scattering (DLS). Total polyphenol content was measured using the Folin–Ciocâlteu method, while antioxidant capacity was evaluated through radical neutralization assays, including ABTS and DPPH, and through iron and copper reduction tests. Antimicrobial activity was tested against Gram-positive and Gram-negative bacteria, as well as *Candida* species. Phenolic compounds and flavonoids from the extracts were essential for the reduction in palladium ions and the stabilization of the nanoparticles. UV/Vis spectroscopy showed a distinct surface plasmon resonance peak, indicating the successful formation of PdNPs. FTIR analysis confirmed the interaction between the bioactive compounds and PdNPs, revealing characteristic peaks of phenolic groups. DLS analysis indicated a hydrodynamic diameter of 63.9 nm for QD-PdNPs, 48 nm for QF-PdNPs, and 63.1 nm for QP-PdNPs, highlighting good dispersion and stability in solution. Although the PdNPs did not exhibit strong antioxidant properties, they demonstrated selective antimicrobial activity, especially against *Staphylococcus aureus* and methicillin-resistant *Staphylococcus aureus* (MRSA). PdNPs also exhibited significant antifungal activity against *Candida krusei*, with a minimum inhibitory concentration (MIC) of 0.63 mg/mL, indicating their ability to compromise fungal cell integrity. This study contributes to developing eco-friendly biosynthesis methods for metallic nanoparticles and underscores the potential of PdNPs in various applications, including in the biomedical field.

## 1. Introduction

Nanotechnology has emerged as an increasingly explored field of research due to its ability to manipulate materials at the nanoscale, imparting unique and valuable properties to them [1]. In this context, the synthesis of metallic nanoparticles has gained significant attention, crucial for a wide range of applications, including biomedicine, environmental protection, and catalysis. However, conventional chemical methods for nanoparticles concern safety and environmental impact [2,3]. In contrast, plant extracts provide an eco-friendly alternative, as they are rich in bioactive compounds that can reduce metal ions while stabilizing the nanoparticles, preventing their aggregation and precipitation. These compounds also influence the nucleation process, leading to the formation of nanoparticles with various morphologies, such as wires, triangles, and cubes [4,5]. Therefore, the biosynthesis of nanoparticles using plant extracts represents a sustainable and environmentally friendly alternative to traditional chemical methods [1].

In recent years, the biosynthesis and application of palladium nanoparticles (PdNPs) have attracted increasing attention from researchers [6]. For instance, Petla et al. described a mechanism by which Pd^2+^ ions are reduced to Pd⁰ by tyrosine, which acts as an electron donor, converting hydroxyl groups into carboxyl groups, as confirmed by FTIR analysis [7]. Similarly, Sheny et al. demonstrated that hydroxyl groups in polyols and glycosides play a crucial role in reducing palladium ions, stabilizing the resulting nanoparticles by forming a protein layer on their surface, as confirmed by FTIR analysis [8]. Although palladium is already used in biomedicine, particularly in dental equipment and the treatment of prostate cancer and choroidal melanoma, the exploration of palladium nanoparticles in nano biomedicine is relatively recent. Due to their high surface-area-to-volume ratio and large surface energy, PdNPs offer significant potential as active catalytic resources in various remediation and biomedical applications [5,9]. Vinodhini et al. [10] used *Allium fistulous*, *Basella alba*, and *Tabernaemontana divaricate* aqueous leaf extracts to synthesize palladium nanoparticles with photocatalytic activity for Congo red dye degradation. Moreover, palladium nanoparticles exhibited antioxidant and antimicrobial properties in a concentration-dependent manner. Furthermore, palladium nanoparticles prepared with *Tabernaemontana divaricate* leaf extract showed antidiabetic activity in vitro, inhibiting the α-amylase enzyme [10].

For the biosynthesis of palladium nanoparticles (PdNPs), extracts from the species of *Quercus* sp. (*Quercus dalechampii*, *Q. frainetto*, and *Q. petraea)* were used. These species are rich in phenolic compounds, flavonoids, and tannins, which are known for their ability to reduce metal ions and stabilize the resulting nanoparticles [11,12,13]. Thus, the extracts from the bark of these oaks contribute both to the reduction in palladium ions and the stabilization of the nanoparticles, ensuring the formation of stable structures. While the biosynthesis of metal nanoparticles using plant extracts has been explored in various studies, to date, no research has investigated the biosynthesis of palladium nanoparticles (PdNPs) using *Quercus* extracts. This research aims to fill that gap by exploring the use of oak bark extracts in the green synthesis of PdNPs and evaluating their bioactive properties.

The primary aim of this research was to biosynthesize palladium nanoparticles (PdNPs) using aqueous extracts from the bark of *Quercus dalechampii*, *Q. frainetto*, and *Q. petraea* and to evaluate their biological activities. To achieve this aim, the research has been focused on three main objectives. The first objective has been to develop and optimize green synthesis methods for PdNPs using aqueous extracts from *Quercus* bark. The second objective has been to thoroughly characterize the synthesized nanoparticles using advanced analytical techniques such as UV/Vis spectroscopy, Fourier-transform infrared spectroscopy (FTIR), and dynamic light scattering (DLS) to determine their relevant physicochemical properties. The third key objective has been to investigate the biological activities of the PdNPs through in vitro testing, focusing on their antioxidant and antimicrobial effects.

## 2. Materials and Methods

### 2.1. Chemicals, Reagents, and Bacterial Strains

For the biosynthesis of PdNPs, PdCl_2_ (≥99.9%) was used, purchased from Sigma-Aldrich, Steinheim, Germany. The following reagents were used for determining total polyphenolic content (TPC) and testing antioxidant potential: Folin–Ciocâlteu reagent (F9252), sodium carbonate (Na_2_CO_3_ 99.5%, 223530), gallic acid (97.5-102.5%, G7384), ABTS reagent (ammonium salt of 2,2′-azino-bis(3-ethylbenzothiazoline-6-sulfonic acid), A1,888), potassium peroxodisulfate (K_2_S_2_O_8_ 99%, 216,224), DPPH (2,2-diphenyl-1-picrylhydrazyl, D9,132), anhydrous sodium acetate (CH_3_COONa ≥99%, S8750), ferric chloride hexahydrate (FeCl_3 ×_ 6H_2_O ≥99%, F2877), TPTZ reagent (2,4,6-tris(2-pyridyl)-S-triazine, T1253), copper(II) chloride (CuCl_2_), and neocuproine (2,9-dimethyl-1,10-phenanthroline), were all supplied by Sigma-Aldrich Chemie GmbH (Taufkirchen, Germany).

For antimicrobial assessments, six bacterial strains and three fungal strains were used, which were obtained from the Department of Microbiology at the “George Emil Palade” University of Medicine, Pharmacy, Science, and Technology in Târgu-Mureș.

The standards employed in both spectrophotometric and UPLC-PDA analyses (including gallic acid, eleutheroside B, chlorogenic acid, catechin, epicatechin, luteolin-3′,7-di-*O*-glucoside, caffeic acid, vanillic acid, luteolin-7-*O*-glucoside, ellagic acid, sinapic acid, taxifolin, and quercetin) all supplied by Sigma-Aldrich (Steinheim, Germany).

### 2.2. Plant Material Collection

**Figure 1:**
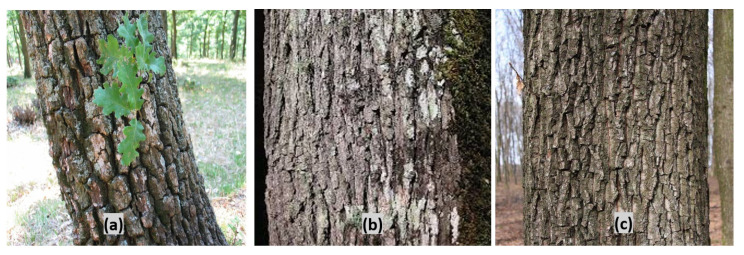
The morphological aspect of *Quercus dalechampii* (**a**), *Quercus frainetto* (**b**), and *Quercus petraea* (**c**).

In May 2021, the bark of *Quercus dalechampii*, *Q. frainetto*, and *Q. petraea* was collected from Zagra, Bistrița-Năsăud County, Romania, a region known for its biodiversity. The material was obtained from residual oak bark, estimated to be from trees aged between 30 and 40 years. Accurate species identification, essential for the reliability of the study, was conducted by Dr. Corneliu Tanase, based on morphological traits. Figure 1 illustrates the morphological differences between the oak species studied: *Quercus dalechampii* has dark brown bark with deep cracks (Figure 1a), *Quercus frainetto* has light gray bark with small square plates (Figure 1b), and *Quercus petraea* has brown bark with fine, subtle fissures (Figure 1c). After collection, the bark was dried at 50 °C for 24 h (Nahita 631 Plus drying oven—Auxilab S.L., Beriáin, Spain) to preserve bioactive compounds, followed by milling of the dried plant material (Pulverisette 15 cutting mill—Fritsch GmbH, Idar-Oberstein, Germany).

### 2.3. Preparation of Aqueous Extract by Quercus sp.

The dried plant material (10 g of bark) was mixed with 100 mL of distilled water in an Erlenmeyer flask. The extraction of bioactive compounds was carried out using an ultrasonic bath [Professional Ultrasonic Cleaner MRC (Beijing, China): AC 150 H, 150 W, 40 kHz, heating power 300 W] for 30 min at a temperature of 60 °C. This method was chosen due to its efficiency in extracting bioactive compounds. After extraction, the mixture was vacuum-filtered to remove solid particles. The supernatant was then centrifuged for 2 min at 10,000 rpm to remove any remaining sediment. The resulting aqueous extracts were immediately used for nanoparticle biosynthesis. For other analyses, the aqueous extracts were lyophilized [BK FD12S freeze-dryer (Biobase Biodustry Co., Ltd., Jinan, China)] and stored in a cool, dry environment, protected from moisture and direct light, and properly labeled. This process resulted in three freeze-dried extracts.

### 2.4. Biosynthesis of PdNPs Using Bioactive Compounds from Quercus sp.

For the biosynthesis of palladium nanoparticles (PdNPs), aqueous extracts of *Quercus dalechampii* and *Quercus frainetto* were combined with a 0.1 mM PdCl_2_ solution in a 1:4 ratio at a pH of 11. Similarly, the aqueous extract from the bark of *Quercus petraea* was mixed with the 0.1 mM PdCl_2_ solution at a 1:9 ratio, maintaining the same pH. The biosynthesis process was carried out at 70 °C for two hours, with the reduction in palladium ions to nanoparticles confirmed by the color change of the mixture from pale yellow to deep brown.

Before obtaining the final conditions for the biosynthesis of palladium nanoparticles (PdNPs), an optimization process was carried out to establish the optimal parameters for biosynthesis. Specifically, different concentrations of PdCl_2_ (0.1, 0.5, 1, and 2 mM), the ratio between the extract and the PdCl_2_ solution (1:4, 1:9, and 1:19), as well as the solution’s pH (initial, 7, and 11) were investigated. Additionally, it was determined that the optimal temperature for the formation of PdNPs is 70 °C. These adjustments were necessary to understand the influence of each parameter on nanoparticle formation and stability.

Continuous monitoring of the biosynthesis process was conducted using UV/Vis spectroscopy, which tracked absorption in the wavelength range of 250–455 nm. This technique allowed real-time observation of the reduction in palladium ions to nanoparticles and was crucial in determining the most effective reaction conditions. After identifying the optimal parameters, the PdNP solutions were lyophilized using a BK-FD12S lyophilizer (Biobase Biodustry Co., Ltd., Jinan, China). The dried nanoparticles (QD-PdNPs, QF-PdNPs, and QP-PdNPs) were stored at +4 °C until further use.

### 2.5. Quantification of Total Phenolic Compounds

The total polyphenol content (TPC) was evaluated for both *Quercus* extracts and nanoparticles formed from the bioactive compounds in these extracts using the Folin–Ciocâlteu method. The Folin–Ciocâlteu reagent was diluted at a 1:10 (*v*/*v*) ratio with distilled water, resulting in a 10% solution. The TPC was assessed using a 96-well plate, where each well received 100 μL of the 10% Folin–Ciocâlteu solution and 20 μL of dried extract or dried nanoparticles (re-dissolved in 70% ethanol at a concentration of 1 mg/mL). The plate was incubated for 3 min at room temperature in the dark to prevent photochemical interference. After this initial incubation, 80 μL of 7.5% Na_2_CO_3_ solution was added, and the plate was incubated for an additional 30 min under the same conditions to allow full color development. Absorbance was measured at 706 nm using a microplate reader (SPECTROstar^®^ Nano, BMG Labtech). Results were quantified based on a gallic acid calibration curve and expressed as milligrams of gallic acid equivalents (GAE) per gram of lyophilized dry matter (mg GAE/g dw.), providing a standardized assessment of polyphenol content.

### 2.6. UPLC-PDA Analysis of Individual Phenolic Constituents

For the identification of phenolic compounds, the initial solutions of extracts and nanoparticles were used, and each sample was filtered through a 0.2 µm nylon membrane before injection. The individual phenolic compounds present in the aqueous *Quercus* extracts (*Quercus dalechampii*, *Q. frainetto*, and *Q. petraea*) and palladium nanoparticles (PdNPs) were analyzed using a HPLC-DAD system, model Flexar FX-10 (Perkin Elmer, Waltham, USA) with the following modules: binary pump, inline degaser, auto sampler with Peltier system, column thermostat and DAD detector. The primary objective was to identify the phenolic compounds responsible for nanoparticle formation.

Chromatographic separation was carried out on a Luna C18 (2) column (3 µm, 150 mm × 4.6 mm) with a flow rate of 1 mL/min. The elution gradient included two phases: phase A (0.1% formic acid) and phase B (acetonitrile), following a specific program to vary the concentration of the mobile phases. Analysis was conducted at 280 nm, using reference standards such as gallic acid, eleutheroside B, chlorogenic acid, catechin, epicatechin, luteolin-3′,7-di-*O*-glucoside, caffeic acid, vanillic acid, luteolin-7-*O*-glucoside, ellagic acid, sinapic acid, taxifolin, and quercetin. Each standard solution was prepared at a concentration of 20 µg/mL, and 20 µL of each standard was injected to ensure accurate identification of the phenolic compounds in the samples.

The identification of these phenolic compounds helped determine those involved in the reduction in palladium ions and the formation of nanoparticles, providing valuable insights for their practical applications.

### 2.7. Characterization of Biosynthesized PdNPs

The physicochemical characterization of nanoparticles is essential for understanding and controlling their properties, a crucial aspect of nanotechnology research and applications.

For the UV/Vis analysis, measurements were performed at a resolution of 1 nm, covering the wavelength range between 250 and 450 nm, using an Analytik Jena Specord 200 Plus spectrophotometer (190—UV/Vis-1100 nm, Analytik Jena AG, Thuringia, Germany). The samples were measured at the initial time point and after 30, 60, 90, and 120 min.

FT-IR spectroscopy was used to investigate compositional changes during the biosynthesis of metallic nanoparticles, using aqueous extracts from the bark of *Quercus* species. This technique enabled the identification of functional groups involved in the biosynthesis process. FT-IR analyses were conducted on lyophilized samples using a spectrophotometer equipped with an ATR crystal in the 400–4000 cm^−1^ range, with a resolution of 4 cm^−1^.

The size of nanoparticles in suspension was determined using dynamic light scattering (DLS), which measured their average hydrodynamic diameter. This technique was adapted from work by Chicea et al. [14].

### 2.8. Quantification of Antioxidant Capacity of PdNPs

The antioxidant capacity of PdNPs was evaluated using four complementary in vitro methods: the DPPH method, the ABTS method, the FRAP method, and the CUPRAC method. In brief, serial dilutions of the samples were prepared in microplates for all four methods, and absorbance readings were taken using a microplate reader (SPECTROstar^®^ Nano Multi-Detection Microplate Reader, BMG Labtech, Ortenberg, Germany), and the results were expressed in mg TE/g dw.

#### 2.8.1. Radical Scavenging Activity

In the case of the DPPH method, 270 μL of methanolic DPPH solution (0.004%) along with 30 μL of nanoparticle solution (1 mg/mL) were added to each well. After 30 min of incubation in the dark, absorbance was measured at 517 nm.

In the case of the ABTS method, for the preparation solution, 10 mL of a 2.15 mM aqueous ABTS solution was mixed with 10 mL of a 1.40 mM potassium persulfate (K_2_S_2_O_8_) aqueous solution. The resulting mixture was incubated at room temperature, in the dark, for 24 h to allow the formation of the ABTS^+^ radical. The solution was then diluted with distilled water to achieve an absorbance of 0.700 (±5%) at 734 nm. For the antioxidant activity assay, 200 μL of the prepared ABTS solution and 20 μL of PdNPs were added to each well. The mixture was incubated for 6 min at room temperature, in the dark. After incubation, the absorbance of the mixture was measured at 734 nm.

#### 2.8.2. Reducing Power

The FRAP (Ferric Reducing Antioxidant Power) assay measures the ability of an antioxidant to reduce ferric ions (Fe^3+^) to ferrous ions (Fe^2+^) in an acidic environment through a single electron transfer (SET) mechanism [15]. In the original FRAP test, tripyridyltriazine (TPTZ) is used as the iron-binding ligand. When the Fe^3+^-TPTZ complex is reduced to its ferrous form (Fe^2+^), it develops an intense blue color and exhibits a maximum absorbance at 593 nm [16]. In short, for the preparation of the FRAP reagent, 10 mL of acetate buffer (pH 3.6; 100 mM) was mixed with 1 mL of 40 mM HCl and 1 mL of 20 mM FeCl_3_ solution. This solution was used to initiate the reduction reaction required for the FRAP assay. Briefly, 175 μL of FRAP reagent was added together with 25 μL of PdNPs in each well. After 30 min of incubation in the dark, absorbance was measured at 593 nm.

The CUPRAC (Cupric Reducing Antioxidant Capacity) assay is a cupric ion-reducing method, considered an improved version of the FRAP test. In this assay, copper is used as the oxidant instead of iron, as in the FRAP method [17]. The assay measures the reducing power of antioxidants to convert cupric ions (Cu^2+^) into cuprous ions (Cu^+^). The chromogenic oxidizing reagent used in the CUPRAC test is neocuproine (Nc; 2,9-dimethyl-1,10-phenanthroline). When an antioxidant reduces the copper (II)-Nc complex to the copper (I)-Nc complex, a yellow/orange color is produced with a maximum absorbance at 450 nm [18]. In short, each sample was mixed with a solution of CuCl_2_, neocuproine, and ammonium acetate at pH 7.0; after 30 min of incubation, the absorbance of the mixture was measured at 450 nm.

### 2.9. Antibacterial Assay

The in vitro antibacterial potential of PdNPs was tested on three Gram-positive bacterial strains [*Staphylococcus aureus* (ATCC 25923), methicillin-resistant *Staphylococcus aureus* (ATCC 43300), *Enterococcus faecalis* (ATCC 29212)] and three Gram-negative bacterial strains [(*Escherichia coli* (ATCC 25922), *Klebsiella pneumoniae* (ATCC 13883), and *Pseudomonas aeruginosa* (ATCC 27853)]. To determine the minimum inhibitory concentration (MIC) of the biosynthesized PdNPs against these bacterial strains, we employed the microdilution method, as previously described [19]. Resazurin was used as an indicator of bacterial growth in highly turbid solutions. A color change in resazurin from blue to pink indicated bacterial presence. The MIC was defined as the last well where no color change in resazurin was observed. From each well where no bacterial growth was detected in the MIC assay, 1 µL of suspension was transferred onto blood agar using a calibrated bacteriological inoculation loop. The minimum bactericidal concentration (MBC) was considered to be the lowest concentration at which no bacterial growth was observed on blood agar.

### 2.10. Antifungal Assay

The in vitro antifungal potential of PdNPs was tested against three fungal strains: *Candida albicans* (ATCC 10231), *Candida krusei* (ATCC 6258), and *Candida auris*, following the methodology described by Coman and collaborators [13]. Standardized fungal cultures with turbidity equivalent to 0.5 on the McFarland scale were prepared using RPMI medium buffered with MOPS and supplemented with 2% glucose. From these cultures, 100 μL were mixed with serial dilutions of each nanoparticle solution to determine the minimum inhibitory concentration (MIC). MIC was defined as the lowest concentration of an antifungal agent necessary to visibly inhibit fungal growth in the medium, determined by observing the absence of fungal growth in the nanoparticle dilutions.

For the determination of the minimum fungicidal concentration (MFC), Sabouraud agar medium was used. The procedure followed a similar methodology to that employed for determining the minimum bactericidal concentration (MBC). MFC was defined as the concentration at which no fungal growth was observed on the Sabouraud agar medium.

### 2.11. Statistical Analysis

All determinations were performed in triplicate, with the final results calculated and expressed as mean ± standard deviation. Statistical analysis of the data was conducted using GraphPad Prism 9.1.0 software, with significant differences between species (*p* < 0.05) evaluated through one-way ANOVA, followed by Tukey HSD post-hoc analysis to assess TPC and antioxidant activity.

## 3. Results and Discussion

### 3.1. Optimization of Biosynthesis Conditions for PdNPs

#### 3.1.1. Influence of Concentration of PdCl_2_

**Figure 2:**
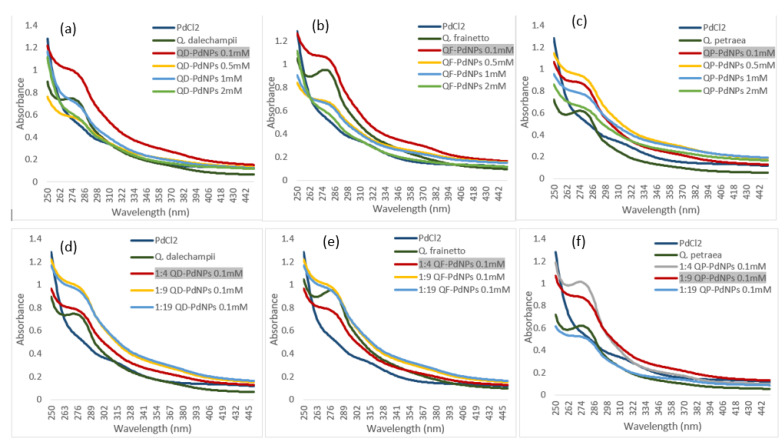
The influence of PdCl_2_ concentration (0.1, 0.5, 1, and 2 mM) [UV/Vis spectra for QD-PdNPs (**a**), QF-PdNPs (**b**), and QP-PdNPs (**c**)] and the extract-to-PdCl_2_ ratio (1:4, 1:9, and 1:19) [UV/Vis spectra for QD-PdNPs (**d**), QF-PdNPs (**e**), and QP-PdNPs (**f**)] on the PdNP formation process.

Optimizing the concentration of the PdCl_2_ solution is crucial for producing PdNPs with precise and reproducible properties. The tested concentrations (0.1 mM, 0.5 mM, 1 mM, and 2 mM) influenced particle size and stability (Figure 2). At 0.5 mM PdCl_2_, higher absorption was observed compared to 0.1 mM, indicating an increased number of nanoparticles. However, the faster decrease in absorption suggests a slight increase in particle size. The absorption spectrum for nanoparticles synthesized at 1 mM PdCl_2_ was similar to that at 0.5 mM but with a curve shape indicating possible aggregation or size increase, affecting uniformity. At 2 mM PdCl_2_, absorption was the lowest among all nanoparticle solutions, suggesting either significant particle growth or pronounced aggregation, leading to reduced stability. In conclusion, lower precursor concentrations, such as 0.1 mM, are more effective in producing small, uniform, and stable PdNPs.

#### 3.1.2. Influence of Extract: PdCl_2_ Solution Ratio

Another important parameter studied in the optimization of PdNPs biosynthesis was the ratio between *Quercus* extracts and the PdCl_2_ solution. Identifying the optimal extract/PdCl_2_ ratio was crucial for achieving the best results with each type of extract tested (Figure 2). For PdNPs synthesized using *Quercus dalechampii* and *Quercus frainetto* extracts, the 1:4 ratio (one part extract to four parts PdCl_2_) proved to be the most effective. UV/Vis spectra showed well-dispersed and stable particles with high absorption in the UV region, indicating efficient biosynthesis. For these extracts, the absorption spectra at the 1:9 and 1:19 ratios were similar, suggesting that increasing the amount of PdCl_2_ did not improve the characteristics of the nanoparticles, likely due to the saturation of reducing and stabilizing agents in the extracts. This can be explained by the rich composition of *Quercus dalechampii* and *Quercus frainetto* extracts in polyphenols and flavonoids, which act as both reducing agents and stabilizers. For nanoparticles synthesized with *Quercus petraea* extract, the optimal ratio was 1:9. The UV/Vis spectrum at this ratio showed significantly higher absorption, indicating smaller particles with superior colloidal stability. This efficiency is attributed to the specific chemical composition of *Quercus petraea* extract, which is rich in phenolic compounds, tannins, and gallic acid, allowing for efficient reduction and stabilization even at higher concentrations of PdCl_2_.

#### 3.1.3. Influence of pH

**Figure 3:**
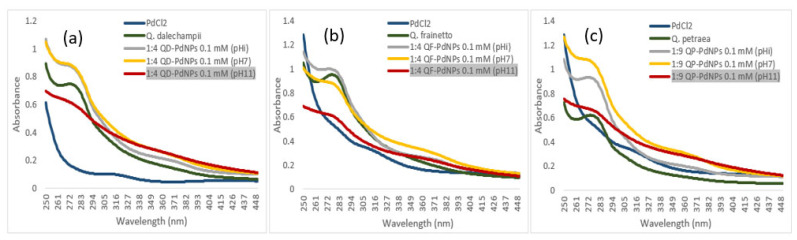
The influence of pH (4, 7, and 11) on the formation process of PdNPs biosynthesized using bioactive compounds from aqueous extracts of the *Quercus* genus [UV/Vis spectra for QD-PdNPs (**a**), QF-PdNPs (**b**), and QP-PdNPs (**c**)].

In the optimization process of PdNP biosynthesis, in addition to adjusting the PdCl_2_ concentration and the ratio between the plant extract and the PdCl_2_ solution, the pH of the solution was also analyzed. According to the results in Figure 3, the UV/Vis spectra for QD-PdNPs, QF-PdNPs, and QP-PdNPs biosynthesized at the initial pH and at pH 7 are nearly identical, indicating that pH adjustments within this range do not significantly affect the size and distribution of the nanoparticles. At pH 11, the spectrum indicates a more efficient biosynthesis and the formation of smaller and more uniform nanoparticles. This can be attributed to the increased reactivity of palladium ions in an alkaline environment, which promotes faster reduction and nucleation [20]. The results showed that a basic pH (with a value of 11) led to the formation of smaller, well-dispersed nanoparticles with improved physicochemical properties, which are consistent with findings from other studies [21,22]. For example, Al-Radadi et al. demonstrated that the biosynthesis of PdNPs is accelerated in basic environments compared to acidic ones, and the efficiency of biosynthesis increases as the concentration of OH^−^ ions rises, i.e., with increased alkalinity of the dispersion medium [23]. This behavior can be explained by the fact that OH^−^ ions facilitate the reduction in palladium ions, thereby accelerating the nucleation process and leading to the formation of smaller and more stable nanoparticles [24].

#### 3.1.4. The Influence of Temperature

Another important factor in the biosynthesis of PdNPs is temperature [25]. Osonga et al. demonstrated that as the reaction temperature increases from 25 °C to 100 °C, the kinetic energy of the molecules involved in the reaction increases proportionally. This rise in kinetic energy favors efficient collisions between molecules, thereby increasing the likelihood of the successful formation of palladium nanoparticles. In their study, the maximum biosynthesis efficiency of PdNPs was observed at 100 °C [26]. In another example, PdNPs biosynthesized using *Origanum vulgare* extract were obtained at a temperature of 90 °C after a reaction time of 2 h [27]. These data suggest that higher temperatures favor the efficient formation of nanoparticles by increasing kinetic energy and, consequently, the frequency of collisions between reactive molecules. In contrast, in our study, the biosynthesis of PdNPs using *Quercus* extracts took place at a lower temperature of 70 °C. This presents a significant advantage for several reasons. First, using a lower temperature can reduce the energy costs associated with the biosynthesis process, making the method more economically sustainable. Second, lower temperatures may help preserve the integrity of the bioactive compounds in the plant extracts, which can be heat-sensitive. This is important because the phenolic compounds and flavonoids in plant extracts not only act as reducing agents but also contribute to the stabilization of the formed nanoparticles. Furthermore, biosynthesis at lower temperatures can reduce the risk of forming aggregated or irregularly sized nanoparticles, thereby contributing to the production of nanoparticles with more uniform and controlled characteristics.

### 3.2. Characterization of PdNPs

#### 3.2.1. UV/Vis Analysis of PdNPs

**Figure 4:**
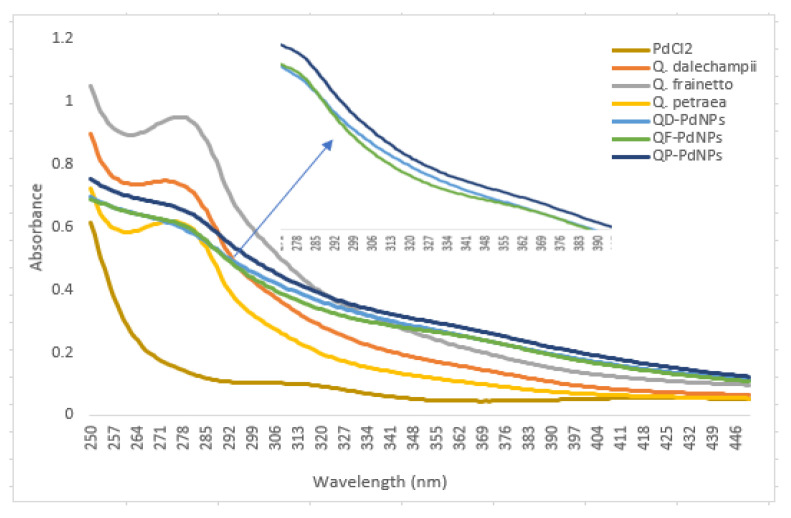
UV/Visible absorption spectra of biosynthesized palladium nanoparticles (QD-PdNPs, QF-PdNPs, and QP-PdNPs).

After the optimization process described above, it was observed that parameters such as the concentration of the PdCl_2_ solution, the ratio between the extract and the PdCl_2_ solution, as well as the pH of the solution, are critical factors in the formation of nanoparticles. Following optimization, the UV/Vis spectra for QD-PdNPs and QF-PdNPs showed differences in the 299–343 nm range, suggesting variations in nanoparticle size (Figure 4); this aspect is also supported by the DLS analysis. The formation of palladium nanoparticles (PdNPs) in the presence of *Quercus* extract is based on a chemical reduction mechanism, where bioactive compounds from the extract act as reducing and stabilizing agents. When PdCl_2_ is dissolved in water, it dissociates, releasing palladium ions (Pd^2+^) and chloride ions (Cl^−^). The palladium ions are in an oxidized state and need to be reduced to form metallic nanoparticles. In a previous study, we demonstrated that *Quercus* extracts contain bioactive compounds such as flavonoids, tannins, polyphenols, and other antioxidant molecules, which can act as reducing agents [13]. These compounds donate electrons and reduce Pd^2+^ ions to neutral palladium atoms (Pd^0^). In the presence of the extract, the palladium ions are reduced to the metallic state through electron transfer from the antioxidant compounds. The formed palladium atoms aggregate to create nuclei, and as more atoms cluster, palladium nanoparticles (PdNPs) develop [28]. As presented in the previous optimization stage, the size and shape of these nanoparticles are influenced by reaction parameters such as the concentration of PdCl_2_, pH, temperature, and the ratio between extract and solution. The bioactive compounds in the extract not only reduce the palladium ions but also stabilize the nanoparticles, preventing their aggregation. Polyphenols and tannins have an affinity for the nanoparticle surface and stabilize them through electrostatic and steric interactions, keeping them dispersed in solution [29].

#### 3.2.2. Fourier Transform Infrared Spectroscopy (FT-IR)

**Figure 5:**
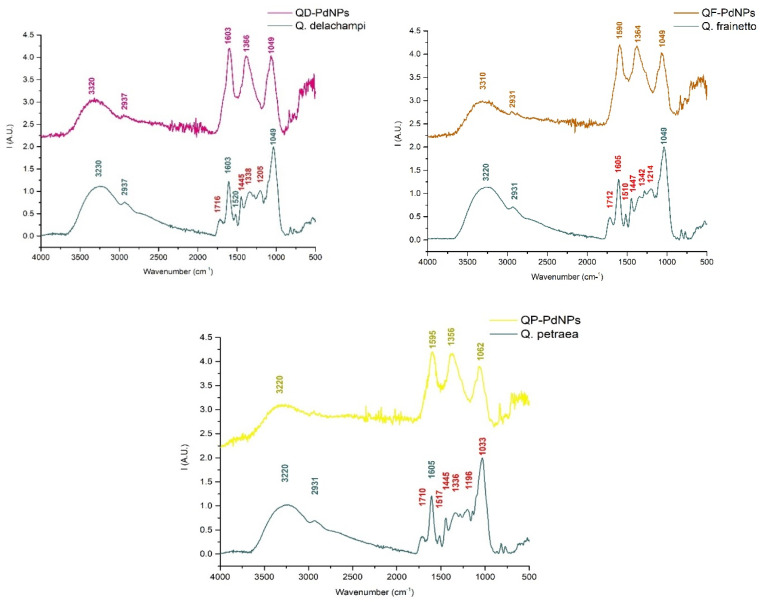
ATR-FTIR spectra of QD-PdNPs (pink line), QF-PdNPs (orange line), QP-PdNPs (yellow line). The bands marked in blue are represented by *Quercus* extracts (*Q. dalechampii*, *Q. frainetto*, *Q. petraea*).

According to Figure 5, FT-IR spectra revealed the functional groups that may act as possible reducing agents in the green synthesis of Pd nanoparticles. The biosynthesized PdNPs exhibit similar chemical groups across all three spectra, highlighted by common peaks. The main peaks are between 3500 and 3200 cm^−1^, with maxima at 3320 cm^−1^ for QD-PdNPs, 3310 cm^−1^ for QF-PdNPs, and 3220 cm^−1^ for QP-PdNPs, and are associated with O-H groups from phenols and polyols [14,30,31]. The action of alcohols as reducing agents in PdNPs formation, a known process in palladium nanoparticle synthesis due to the oxidant effect of Pd(II) [32,33], is sustained by the observation of a lower intensity peak in the case of PdNPs compared to each extract. Therefore, a decrease in phenol content in the NP samples is suggested. The interaction of extracts with Pd ions and NP formation is also sustained by a decrease in peak intensity at 2937 cm^−1^ for QD-PdNPs and 2931 cm^−1^ for QF-PdNPs, bonds associated with C/H vibrations from polysaccharides [34]. These bonds confirm that PdNPs can adsorb hydroxyl groups and other stabilizing groups.

In the lower regions of the FTIR spectra, notable differences were observed between the initial extracts and the biosynthesized PdNPs, sustaining the formation of PdNPs but also showing different processes that occurred during the synthesis.

For instance, the absence of each green synthesizes PdNPs from 1716 cm^−1^ in the case of *Q. delachampi*, 1712 cm^−1^ in *Q. frainetto,* and 1710 cm^−1^ in *Q. petraea*, corresponding to the carbonyl groups (C=O stretch) [35], is associated with the reaction of carboxylic acids in the basic media used in the synthesis process in which carboxylate salts were obtained. These reactions of carboxylic acids are also sustained by the absence of 1205 cm^−1^ for QD-PdNPs, 1214 cm^−1^ for QF-PdNPs, and 1196 cm^−1^ for QP-PdNPs, bonds corresponding to C-O stretch vibration in carboxyl esters [35,36,37]. As *Querques* extracts are rich in phenols, tannins, and flavanols, a possible reduction mechanism is based on the oxidation of Pd(II) using alcohols and a basic pH medium, which favors the appearance of stabilizing carboxylate esters at the surface of the nanoparticle [8,38]. Furthermore, the extract chemical groups absorption at the NPs surface is lately suggested by the absence of 1445 cm^−1^ bound for QD-PdNPs/QP-PdNPs and 1447 cm^−1^ for QF-PdNPs associated with C=C from aromatic compounds, thus reflecting a strong interaction of these compounds with the metallic salt during each synthesis [39].

All these differences between the extracts and their synthesized NPs strongly suggest a high number of compounds involved in the synthesis and absorbed by the NP’s surface while they are stabilizing in solution.

#### 3.2.3. DLS Analyses of PdNPs

DLS (dynamic light scattering) analysis was used to measure the hydrodynamic diameters of palladium nanoparticles (PdNPs) obtained from different oak extracts. The nanoparticles QD-PdNPs (63.9 ± 8.3 nm) and QP-PdNPs (63.1 ± 8.2 nm) exhibit similar sizes, suggesting that extracts from *Quercus dalechampii* and *Quercus petraea* generate nanoparticles with comparable organic layers, influencing their stability and final size. In contrast, QF-PdNPs (48.0 ± 6.2 nm) show smaller dimensions, indicating that the *Quercus frainetto* extract produces nanoparticles with a thinner or structurally different organic layer, promoting more efficient dispersion and enhanced stability in suspension. The sizes of the palladium nanoparticles obtained in this study align with the literature, which demonstrates that PdNPs sizes vary significantly depending on the plant extract used and other optimization factors in the synthesis process. Overall, the values observed in this study and those reported in the literature generally range between 30 and 70 nm, highlighting the specific influence of each extract’s chemical composition on the reduction and stabilization process of the nanoparticles [40,41,42].

### 3.3. Phytochemical Profile of PdNPs Biosynthesized from Quercus sp. Extracts

According to the results published in another article, the total polyphenol content (TPC) in the extracts of *Quercus dalechampii*, *Quercus frainetto*, and *Quercus petraea* was 402.23 mg/mL, 437.68 mg/mL, and 325.03 mg/mL, respectively [13]. After the biosynthesis of palladium nanoparticles, the remaining TPC in the PdNPs solutions decreased significantly, reaching 50.65 mg/mL for QD-PdNPs, 44.37 mg/mL for QF-PdNPs, and 30.38 mg/mL for QP-PdNPs (Table 1). The observed difference in the total phenolic content (TPC) between the plant extract and the TPC level after the formation of PdNPs suggests the involvement of polyphenols and flavonoids in the biosynthesis process of palladium nanoparticles [43]. These compounds not only act as reducing agents, transforming metal ions into zero-valent metallic nanoparticles, but they also contribute to the colloidal stability of the nanoparticles. Polyphenols and flavonoids ensure stability by forming electrostatic interactions and hydrogen bonds with the surface of the nanoparticles, thus preventing their aggregation [44,45,46]. This observation was confirmed in the present study through FT-IR and HPLC analyses, which revealed the presence of functional groups associated with these compounds on the nanoparticle surfaces.

**Figure 6:**
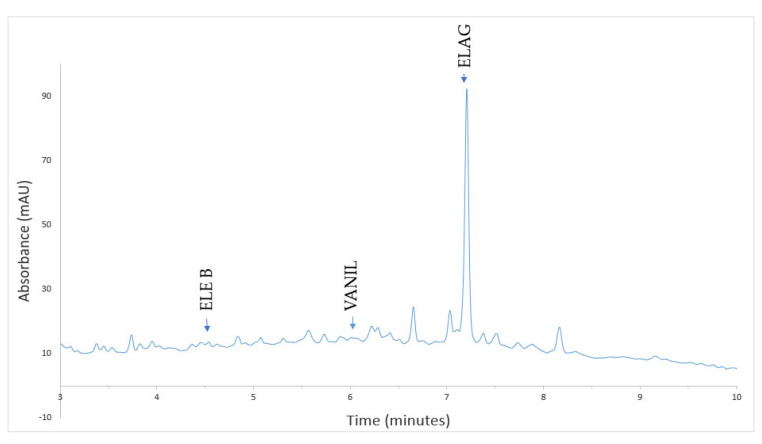
HPLC chromatogram of PdNPs using *Quercus dalechampii* (QD-PdNPs) at 280 nm.

**Figure 7:**
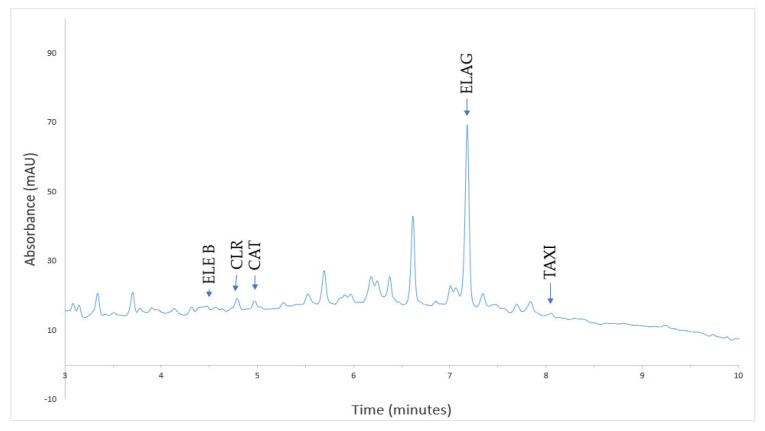
HPLC chromatogram of PdNPs using *Quercus frainetto* (QF-PdNPs) at 280 nm.

**Figure 8:**
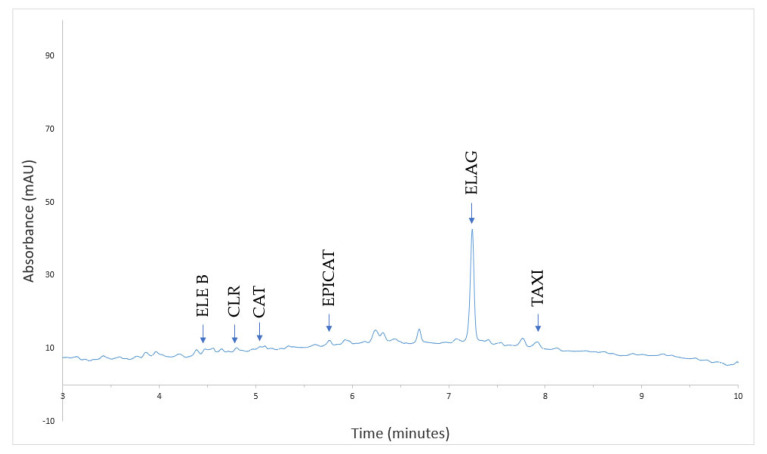
HPLC chromatogram of PdNPs using *Quercus petraea* (QP-PdNPs) at 280 nm.

In a previously published study, HPLC analysis of phenolic compounds demonstrated that in *Quercus dalechampii*, all 13 analyzed compounds were found in the solution. However, in *Quercus frainetto*, vanillic acid and sinapic acid were not detected, and in *Quercus petraea*, caffeic acid and luteolin-7-*O*-glucoside were not identified [13]. During the biosynthesis of PdNPs, the phenolic compounds from *Quercus* extracts were consumed in various chemical processes. Some of these compounds acted as reducing agents, donating electrons to reduce palladium ions to their metallic form. Other phenolic compounds played a role as stabilizers, adsorbing onto the surface of the nanoparticles and preventing their aggregation [46]. After the biosynthesis of PdNPs, three compounds were identified in QD-PdNPs: eleutheroside B, vanillic acid, and ellagic acid (Figure 6). In QF-PdNPs, five compounds were identified: eleutheroside B, chlorogenic acid, catechin, ellagic acid, and taxifolin (Figure 7). In QP-PdNPs, six compounds were identified: eleutheroside B, chlorogenic acid, catechin, epicatechin, ellagic acid, and taxifolin (Figure 8). The compounds that remained in the nanoparticles, though in smaller amounts, played an essential role in stabilizing the nanoparticles and maintaining their physicochemical properties. These compounds can help prevent nanoparticle aggregation and may influence their reactivity and stability in suspension.

### 3.4. Antioxidant Activity

Antioxidant activity was evaluated using various methods, including free radical scavenging capacity (DPPH, ABTS) and metal ion reduction capacity (FRAP and CUPRAC). The results show variations depending on the testing method applied. According to the data presented (Table 2), antioxidant activity values for PdNPs are lower compared to *Quercus* extracts, regardless of the evaluation method used. The antioxidant activity of the extracts has been detailed in another study [13]. Different letters following the values indicate statistically significant differences between the PdNP samples and the *Quercus* extracts. For instance, the antioxidant activity measured by the DPPH method for the QD extract is 2050 mg TE/g dw., whereas for QD-PdNPs, it decreases to 53.60 mg TE/g dw. This consistent trend suggests that incorporating *Quercus* extracts onto palladium nanoparticles significantly reduces antioxidant activity. Several factors can explain the differences in antioxidant activity between pure *Quercus* extracts and PdNPs loaded with these extracts. The first factor is the stability of phenolic compounds. The total phenolic content (TPC) is also lower in PdNPs compared to pure extracts. This may indicate partial oxidation or inactivation of phenolics during the PdNP synthesis process, potentially leading to reduced antioxidant capacity, given that phenolics are the primary compounds responsible for antioxidant activity [47]. The second factor involves interactions between PdNPs and active compounds [48]. Palladium nanoparticles may directly interact with antioxidant compounds in the *Quercus* extracts, influencing their reactivity. Palladium, due to its catalytic nature, may induce structural changes or diminish the antioxidant activity of the bioactive compounds in the extract [49].

The results suggest that the lower antioxidant activity values observed for PdNPs appear to be directly related to the lower TPC values. This finding indicates that the antioxidant activity of PdNPs is more influenced by the phenolic content in the extract than by the inherent properties of palladium nanoparticles. Generally, phenolic compounds are the main contributors to antioxidant activity in natural extracts [50], and the decrease in TPC in PdNPs samples largely explains the reduced antioxidant activity.

### 3.5. Antibacterial Activity

The results in Table 3 show a significant difference in antibacterial efficacy between *Quercus* extracts (QD, QF, QP) and palladium nanoparticles (PdNPs). This effect also correlates with observations on antioxidant activity, where a reduction in total polyphenol content (TPC) led to lower antioxidant activity in the case of PdNPs. This reduction in TPC also explains the higher values of MIC (Minimum Inhibitory Concentration) and MBC (Minimum Bactericidal Concentration) for PdNPs compared to the extracts. The antibacterial activity of *Quercus* extracts was previously detailed in another article, providing a basis for comparison with the increased efficacy observed in the case of palladium nanoparticles derived from these extracts [13].

Regarding the antibacterial activity against *Staphylococcus aureus*, the QF extract had an undetermined MBC at the analyzed concentration (>5 mg/mL), indicating low bactericidal efficiency. In contrast, QF-PdNPs had an MBC of 2.5 mg/mL, demonstrating a significant improvement in bactericidal effect. This result may be attributed to the nanoparticles’ capacity to penetrate the bacterial cell wall more effectively, leading to more efficient bactericidal activity.

For the MRSA strain (methicillin-resistant *Staphylococcus aureus*), the QD, QF, and QP extracts failed to completely eradicate the bacteria (MBC > 5 mg/mL). In contrast, the combinations with PdNPs (QD-PdNPs, QF-PdNPs, QP-PdNPs) showed strong bactericidal effects, with an MBC of 2.5 mg/mL. As a strain resistant to conventional antimicrobial treatments, MRSA requires more potent antimicrobial agents, and palladium nanoparticles appear to enhance the antibacterial effect of the extracts. This indicates that PdNP solutions have strong bacteriostatic and bactericidal effects at the analyzed concentrations.

For *Enterococcus faecalis* and *Escherichia coli*, all solutions, both with PdNPs and extracts, showed MIC and MBC values > 5.00 mg/mL, suggesting a high resistance of these bacteria to the tested solutions. This suggests that the nanoparticles failed to significantly improve the antibacterial effect on these bacteria, possibly due to their resilient cellular structure or well-developed defense mechanisms.

In the case of *Klebsiella pneumoniae*, the QD and QF extracts had MBC values > 5 mg/mL, suggesting low bactericidal efficiency. In contrast, the QD-PdNPs and QF-PdNPs combinations had an MBC of 2.5 mg/mL, indicating an improvement in bactericidal effect through the use of PdNPs.

For *Pseudomonas aeruginosa*, the extracts displayed bactericidal effects, but the PdNP solutions did not show bactericidal activity at the analyzed concentrations (MBC > 5 mg/mL). *Pseudomonas aeruginosa* is known for being highly resistant to antimicrobial treatments, and the lack of observed efficacy for palladium nanoparticles suggests that these solutions are not potent enough to eliminate this bacterium.

These observed differences between the extracts and the PdNPs obtained from these extracts can be explained by the synergy between palladium nanoparticles and the bioactive compounds in *Quercus* extracts, which may lead to more efficient bactericidal activity even at lower concentrations. PdNPs can increase the permeability of the bacterial cell wall, thus facilitating the penetration and more effective action of the bioactive compounds from the extract, resulting in an enhanced capacity to eliminate bacteria [51].

Regarding the antibacterial activity of biosynthesized PdNPs, Al-Fakeh et al. demonstrated that PdNPs obtained using propolis had no effect on the fungus *Aspergillus niger* but generated the largest inhibition zone against *E. coli* and showed moderate activity against *Staphylococcus aureus* [52]. These findings are supported by other studies that have also shown that PdNPs exhibit the strongest antibacterial activity against *E. coli* [53,54]. PdNPs biosynthesized from *Bauhinia variegata* extract, with sizes ranging from 2 to 8 nm and irregular shapes, displayed notable antibacterial activity against *Bacillus subtilis* and significant antifungal activity against *Candida albicans*. Additionally, these nanoparticles showed antitumor potential against the MCF-7 breast cancer cell line [55]. Anand et. al. showed that PdNPs biosynthesized from *Moringa oleifera* flower extract, with sizes between 10 and 50 nm and a spherical shape, demonstrated effective antibacterial activity against *Enterococcus faecalis* and exhibited cytotoxic activity against human lung carcinoma cells (A549) and peripheral lymphocytes [56]. In another study, Surendra et al. showed that PdNPs obtained from *Moringa oleifera* flower extract had stronger antibacterial activity against *Staphylococcus aureus* compared to *E. coli* and did not show toxicity toward red blood cells (RBC) [57].

### 3.6. Antifungal Activity

The results in Table 4 illustrate the antifungal efficacy of various *Quercus* extracts (QD, QF, and QP) and palladium nanoparticles (PdNPs) derived from these extracts against three pathogenic fungi species: *Candida albicans*, *Candida krusei*, and *Candida auris*. The antifungal activity of the extracts was previously detailed in another article [13]. The data presented include values for the minimum inhibitory concentration (MIC) and minimum fungicidal concentration (MFC).

*Candida albicans* and *Candida auris* were resistant to all tested solutions, suggesting that these species require higher concentrations for effective antifungal activity. In contrast, for *Candida krusei*, a clear improvement in antifungal activity is observed with PdNPs compared to the extracts. Among the studied solutions, the most effective was QP-PdNPs, which had the lowest MIC of 0.63 mg/mL, indicating that palladium nanoparticles significantly enhanced fungal inhibition. QD-PdNPs showed a notable improvement compared to the *Quercus dalechampii* extract, with an MIC of 2.5 mg/mL and an MFC of 5 mg/mL. These results suggest that the addition of palladium nanoparticles improved the inhibition capacity, although the complete fungicidal effect remained at the same level as the extract. Similarly, QF-PdNPs showed enhanced inhibition with a MIC of 1.25 mg/mL, but the MFC remained > 5 mg/mL, indicating a limited effect on fully eradicating the fungus.

These observed improvements in antifungal activity against *Candida krusei* can be explained by the synergy between palladium nanoparticles and the bioactive compounds in *Quercus* extracts. PdNPs may increase the permeability of the fungal cell wall, thereby facilitating the penetration and action of active compounds from the extract, leading to more effective inhibition of fungal growth [58].

## 4. Conclusions

The obtained results suggest that extracts from *Quercus* bark can be used as suitable reducing agents in the synthesis of PdNPs. Parameters such as precursor concentration, extract/PdCl_2_ ratio, and solution pH had a significant impact on the size and stability of PdNPs. The best results were obtained at lower PdCl_2_ concentrations (0.1 mM) and under basic pH conditions. Additionally, the phenolic compounds and flavonoids in the plant extracts played an essential role in reducing palladium ions and stabilizing the nanoparticles. Although the PdNPs had small sizes and a large interaction surface, their antioxidant activity was limited by the absence of antioxidant compounds in their structure. Regarding antimicrobial activity, PdNPs demonstrated selective efficacy against *Staphylococcus aureus* and MRSA bacteria and notable antifungal activity against *Candida krusei*, with a possible mechanism based on the generation of reactive oxygen species (ROS).

Future research will focus on examining how these nanoparticles interact with cell membranes and other biological structures, highlighting the mechanisms by which they affect bacterial and fungal cells. Additionally, the long-term impact of nanoparticles on various cell types and living organisms will be assessed, including studies on their biodistribution, metabolism, and elimination. These findings open up new perspectives for nanoparticle applications, ranging from antimicrobial treatments and medical products to drug delivery systems. The ultimate goal is to translate this research into practical applications through testing and establishing guidelines for their use in various applications, including in the biomedical field.

## Figures and Tables

**Table 1:** Retention time of phenolic compounds identified and quantified in QD-PdNPs, QF-PdNPs, and QP-PdNPs, expressed in μg/mL, detected at 280 nm.

Compound	Abbreviation	Retention Time (min)	QD-PdNPs	QF-PdNPs	QP-PdNPs
Eleutheroside B	ELE B	4.5	0.30	0.33	0.06
Chlorogenic acid	CLR	4.8	-	0.05	0.02
Catechin	CAT	5.0	-	0.04	0.05
Epicatechin	EPICAT	5.7	-	-	0.59
Vanillic acid	VANIL	6.0	0.11	-	-
Ellagic acid	ELAG	7.2	1613.85	1021.38	588.43
Taxifolin	TAXI	8.0	-	0.05	0.03

**Table 2:** The estimation of total phenolic content was based on the Folin–Ciocalteu method, and the antioxidant activity of the PdNPs and extract was evaluated.

Sample Code	TPC mg/mL	DPPH mg TE/g dw.	ABTS mg TE/g dw.	FRAP mg TE/g dw.	CUPRAC mg TE/g dw.
QD	402.24 ± 14.29 ^a^	2050 ± 24.74 ^a^	2450.24 ± 136.45 ^a^	2350.31 ± 21.33 ^a^	810.69 ± 15.91 ^a^
QD-PdNPs	50.65 ± 1.59 ^b^	53.60 ± 2.48 ^b^	109.28 ± 1.18 ^b^	40.04 ± 0.62 ^b^	99.73 ± 0.61 ^bf^
QF	437.68 ± 12.92 ^c^	1424.00 ± 32.49 ^c^	1086.96 ± 170.76 ^c^	1578.17 ± 19.25 ^c^	844.89 ± 7.43 ^c^
QF-PdNPs	44.37 ± 1.42 ^b^	65.14 ± 1.34 ^b^	125.02 ± 1.28 ^b^	46.80 ± 0.10 ^b^	123.53 ± 10.07 ^b^
QP	325.03 ± 10.43 ^d^	114.79 ± 1.28 ^d^	1852.57 ± 86.57 ^d^	122.33 ± 0.88 ^d^	138.07 ± 0.62 ^d^
QP-PdNPs	30.39 ± 1.94 ^b^	45.28 ± 0.89 ^b^	89.54 ± 1.18 ^b^	33.41 ± 0.27 ^b^	76.12 ± 2.82 ^ef^

The results are expressed as the mean of three determinations ± standard deviation. Statistical differences between species were evaluated using one-way ANOVA, followed by post-hoc Tukey HSD analysis. Significant differences are indicated above the error bars by lowercase letters (a–f). QD—*Quercus dalechampii*, QD-PdNPs-PdNPs biosynthesized with *Quercus dalechampii*, QF—*Quercus frainetto*, QF-PdNPs-PdNPs biosynthesized with *Quercus frainetto*, QP—*Quercus petraea*, QP-PdNPs-PdNPs biosynthesized with *Quercus petraea*, TPC, total phenolic content; DPPH, free radical scavenging activity against 2,2-diphenyl-1-picrylyl radicals; TE, Trolox equivalents; ABTS, free radical scavenging activity against 2,2′-azino-bis(3-ethylbenzothiazoline-6-sulfonate); TE, Trolox equivalents; FRAP, ferric reducing antioxidant power; Fe(II) chelating capacity, ferrous ion chelating capacity; CUPRAC, cupric reducing antioxidant capacity.

**Table 3:** Antimicrobial activity of PdNPs of tested bacteria.

Pathogenic Bacteria	ATCC No	PdNPs Tested Solution	MIC	MBC
			mg/mL	mg/mL
*Staphylococcus aureus*	25923	QD	0.62	2.50
		QD-PdNPs	2.50	2.50
		QF	0.62	>5.00
		QF-PdNPs	2.50	2.50
		QP	1.25	5.00
		QP-PdNPs	2.50	5.00
		QD	0.62	>5.00
MRSA	43300	QD-PdNPs	2.50	2.50
		QF	0.62	>5.00
		QF-PdNPs	2.50	2.50
		QP	0.31	>5.00
		QP-PdNPs	2.50	2.50
		QD	>5.00	>5.00
*Enterococcus faecalis*	29212	QD-PdNPs	>5.00	>5.00
		QF	>5.00	>5.00
		QF-PdNPs	>5.00	>5.00
		QP	>5.00	>5.00
		QP-PdNPs	>5.00	>5.00
		QD	>5.00	>5.00
*Escherichia coli*	25922	QD-PdNPs	>5.00	>5.00
		QF	>5.00	>5.00
		QF-PdNPs	>5.00	>5.00
		QP	>5.00	>5.00
		QP-PdNPs	>5.00	>5.00
		QD	0.62	>5.00
*Klebsiella pneumoniae*	13883	QD-PdNPs	2.50	2.50
		QF	0.62	>5.00
		QF-PdNPs	2.50	2.50
		QP	0.31	0.62
		QP-PdNPs	2.50	2.50
		QD	2.50	5.00
*Pseudomonas aeruginosa*	27853	QD-PdNPs	>5.00	>5.00
		QF	1.25	5.00
		QF-PdNPs	>5.00	>5.00
		QP	0.62	2.50
		QP-PdNPs	>5.00	>5.00

Notes: MRSA—methicillin-resistant *Staphylococcus aureus*, QD—*Quercus dalechampii*, QD-PdNPs-PdNPs biosynthesized with *Quercus dalechampii*, QF—*Quercus frainetto*, QF-PdNPs-PdNPs biosynthesized with *Quercus frainetto*, QP—*Quercus petraea*, QP-PdNPs-PdNPs biosynthesized with *Quercus petraea*, MIC—minimum inhibitory concentration, MBC—minimum bactericidal concentration.

**Table 4:** Antimicrobial activity of PdNPs of tested fungi.

Pathogenic Bacteria	ATCC	PdNPs Solution	MIC	MFC
			mg/mL	
		QD	>5.00	>5.00
*Candida albicans*	10231	QD-PdNPs	>5.00	>5.00
		QF	>5.00	>5.00
		QF-PdNPs	>5.00	>5.00
		QP	>5.00	>5.00
		QP-PdNPs	>5.00	>5.00
		QD	5.00	>5.00
*Candida krusei*	6258	QD-PdNPs	2.50	>5.00
		QF	5.00	>5.00
		QF-PdNPs	1.25	>5.00
		QP	2.50	>5.00
		QP-PdNPs	0.63	>5.00
		QD	>5.00	>5.00
*Candida auris*		QD-PdNPs	>5.00	>5.00
		QF	>5.00	>5.00
		QF-PdNPs	>5.00	>5.00
		QP	>5.00	>5.00
		QP-PdNPs	>5.00	>5.00

Notes: QD—*Quercus dalechampii*, QD-PdNPs—PdNPs biosynthesized with *Quercus dalechampii*, QF—*Quercus frainetto*, QF-PdNPs—PdNPs biosynthesized with *Quercus frainetto*, QP—*Quercus petraea*, QP-PdNPs—PdNPs biosynthesized with *Quercus petraea*, MIC—minimum inhibitory concentration, MFC—minimum fungicidal concentration.

## Data Availability

Data is contained within the article.
